# Nanoparticle-Based Rifampicin Delivery System Development

**DOI:** 10.3390/molecules26072067

**Published:** 2021-04-03

**Authors:** Marjan Motiei, Luis Pleno de Gouveia, Tomáš Šopík, Robert Vícha, David Škoda, Jaroslav Císař, Reza Khalili, Eva Domincová Bergerová, Lukáš Münster, Haojie Fei, Vladimír Sedlařík, Petr Sáha

**Affiliations:** 1 Centre of Polymer Systems, University Institute, TBU, tr. Tomase Bati 5678, 76001 Zlin, Czech Republic; sopik@utb.cz (T.Š.); dskoda@utb.cz (D.Š.); jcisar@utb.cz (J.C.); domincova_bergerova@utb.cz (E.D.B.); munster@utb.cz (L.M.); haojie@utb.cz (H.F.); sedlarik@utb.cz (V.S.); saha@utb.cz (P.S.); 2 iMed.ULisboa, Faculty of Pharmacy, Universidade de Lisboa, 169-003 Lisbon, Portugal; lgouveia@campus.ul.pt; 3 Department of Chemistry, Faculty of Technology, TBU, Vavrečkova 275, 76001 Zlín, Czech Republic; rvicha@utb.cz; 4 Department of Paediatrics and Inherited Metabolic Disorders, First Faculty of Medicine, Charles University and General University Hospital in Prague, Ke Karlovu 455/2, 12808 Prague 2, Czech Republic; rezakhalili77@gmail.com

**Keywords:** rifampicin, polyelectrolyte nanoparticles, ascorbic acid, alkaline pH

## Abstract

The alkaline milieu of chronic wounds severely impairs the therapeutic effect of antibiotics, such as rifampicin; as such, the development of new drugs, or the smart delivery of existing drugs, is required. Herein, two innovative polyelectrolyte nanoparticles (PENs), composed of an amphiphilic chitosan core and a polycationic shell, were synthesized at alkaline pH, and in vitro performances were assessed by ^1^H NMR, elemental analysis, FT-IR, XRD, DSC, DLS, SEM, TEM, UV/Vis spectrophotometry, and HPLC. According to the results, the nanostructures exhibited different morphologies but similar physicochemical properties and release profiles. It was also hypothesized that the simultaneous use of the nanosystem and an antioxidant could be therapeutically beneficial. Therefore, the simultaneous effects of ascorbic acid and PENs were evaluated on the release profile and degradation of rifampicin, in which the results confirmed their synergistic protective effect at pH 8.5, as opposed to pH 7.4. Overall, this study highlighted the benefits of nanoparticulate development in the presence of antioxidants, at alkaline pH, as an efficient approach for decreasing rifampicin degradation.

## 1. Introduction

Rifampicin (RIF), a hydrophobic zwitterionic antibiotic with a basic (pKa 7.9) and an acidic (pKa 1.7) moiety [1], is commonly prescribed as the strongest bactericidal for the treatment of various infections caused by surface-adhering microorganisms because of its broad-spectrum activity against most Gram-positive, and some Gram-negative, bacteria. Bactericidal activity is performed by hindering the gene transcription using inhibition of DNA-dependent RNA polymerase [2,3]. However, the use of this semisynthetic antibiotic is limited due to various drawbacks, such as poor solubility, low bioavailability [4], modification of skin microbiome, the emergence of RIF resistance, and hepatotoxicity [5]. It has been reported that RIF is chemically unstable at both acidic and alkaline media, as it is hydrolyzed to 3-formylrifamycin SV and 1-amino-4-methylpiperazine in acidic environments [1], and autoxidized to different oxidized species, such as inactive RIF quinone (RIFQ) in alkaline solutions [6]. Therefore, it is necessary to suggest comprehensive strategies for improving drug protection from the external environment during manufacturing and throughout the medicine’s shelf-life.

One of the strategies for overcoming these limitations is utilizing nanoparticles to improve drug delivery platforms, as well as antioxidants to counteract the effects of oxidative stress [7]. Rajaram et al. confirmed that the co-administration of ascorbic acid (ASC) and RIF increased RIF bioavailability by decreasing in situ RIF degradation in acidic conditions [8]. Despite both in vitro and in vivo experiments demonstrating that ASC acted as an antioxidant and an anti-inflammatory factor, without any effect on the antibacterial activity of RIF [9,10], only a few studies used ASC in the release or dissolution medium [11]. However, various research groups have made attempts to exploit nanoparticles, such as dendrimers [12], polymeric nanoparticles [5,11,13], solid lipid nanoparticles [14], gold nanorods [15], and silica nanoparticles [16], for RIF delivery, but no study has directly specified nanoparticles for alkaline conditions. 

Chronic nonhealing wounds are one of the highly proteolytic environments with elevated alkaline pH. Upon injury of healthy skin, the leakage of microvessels increases the pH of the wound surface to physiologic pH (7.4), which rises gradually with increasing depth [17]. During the healing process, the wound environment may become predominately alkaline in chronic nonhealing wounds, within the range of 7.15–8.9, due to the presence of ammonia liberated from urea [18]. Wounds with a high alkaline pH show a low healing rate due to higher risk of polymicrobial infections [19] and hinderance of the antiseptic’s efficacy [18]. Therefore, in the present scenario, we propose that the innovative polyelectrolyte nanoparticles (PENs), comprised of amphiphilic chitosan core for encapsulating RIF, enhance their bioavailability by reducing their interaction with the alkaline microenvironment of chronic wounds, and that the polycationic shell synthesized at alkaline pH preserves the PENs integrity during direct contact with the alkaline environment. 

Chitosan (CS), a linear amino-polysaccharide composed of glucosamine and *N*-acetylglucosamine, is used as an ingredient in pharmaceutical formulations due mainly to its safety and additional desirable characteristics, including biocompatibility, biodegradability, bacteriostaticity, and low immunogenicity [20]. To induce the self-assembly of the core structure and enhance the potential for entrapment of hydrophobic RIF, CS was modified with two hydrophobic amino acids, including alanine (A) with an aliphatic hydrocarbon side chain, and tryptophan (W) with a large rigid aromatic side chain. It was proposed that these different hydrophobic moieties would affect the physicochemical characteristics and release rate of RIF-loaded PENs modified by A (APENs) and W (WPENs). 

The shell was composed of a positively charged polymer, polyethyleneimine (PEI), with three kinds of amine groups partially protonated at physiological and endosomal pH. Therefore, decoration of the nanoparticles with PEI shell enhances endosomal escape by its intrinsic “proton sponge effect” [21], and, simultaneously, demonstrates a wide array of electrostatic interactions with negatively dextran sulphate (DS) coating the core. DS, negatively charged polysaccharide, is a biodegradable and biocompatible polyanion which interacts electrostatically with the positive amino groups of CS and PEI [22]. It is expected that the strong interactions among these layers cause high stability of the nanoparticles, and, consequently, induce more compact and stable nanostructures. 

Overall, this study was initiated with the main goal of circumventing the main causes of poor results obtained during RIF assays, and to protect it against the harsh environment of chronic wounds. The innovation of the present work was to synthesize polyelectrolyte nanosystems composed of an inner amphiphilic CS core modified by A and W, a polyanionic cross-linker DS, and a PEI shell with alkaline pH, and to design the experimental conditions for evaluation of the simultaneous protective effects of PENs and antioxidants on RIF against alkaline environments. It was expected that the different amphiphilic core structures could affect the physicochemical properties of PENs, which were fully characterized by Fourier-transform infrared spectroscopy (FT-IR), elemental analysis, proton nuclear magnetic resonance (^1^H NMR), X-ray diffraction (XRD), differential scanning calorimetry (DSC), Dynamic light scattering (DLS), scanning electron microscopy (SEM), and transmission electron microscopy (TEM). We also proposed that the engineered nanoparticles and ASC would synergistically improve the release and degradation rate of RIF at alkaline pH, which was analyzed in different release media by UV/Vis spectrophotometry and HPLC. 

## 2. Results and Discussion

### 2.1. Evaluation of Size, Morphology, and ζ-Potential of PENs

**Figure 1:**
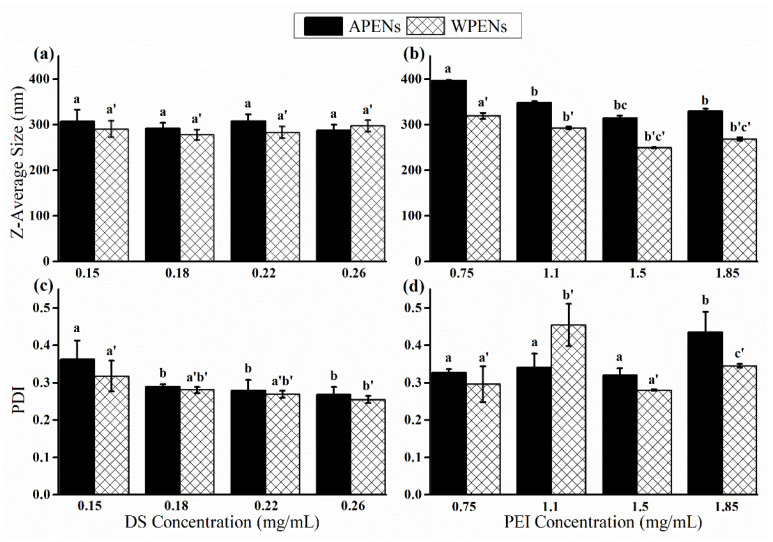
The effect of DS and PEI concentration on z-average size (**a**,**b**) and PDI (**c**,**d**) of the PENs. Different letters of a, b and c indicate significant differences between the z-average size and PDI of APENs at different concentrations of DS and PEI, but a’, b’ and c’ are related to significant differences in WPENs (*p* value < 0.05).

The physicochemical properties of PENs are influenced by the strength of ionic interactions among the layers, the structural features of grafted moieties and cargo, and the substitution degree [23]. Therefore, different concentrations of DS and PEI were added to the constant concentration of CS to obtain small nanoparticles with a relatively narrow size distribution. The smallest size and relatively narrow distribution of APENs (291.4 ± 12.5 nm and 0.29 ± 0.00) and WPENs (277.2 ± 11.5 nm and 0.28 ± 0.00) were observed at the optimal concentration of DS, in the range of 0.18 mg/mL (Figure 1). It was expected that WPENs show larger z-average size than APENs due to the large bulky aromatic side chain of W, as opposed to the small aliphatic side chain of A, which affects the conformation of the surrounding structure [24,25]. Nonetheless, this study showed apparently conflicting evidence regarding the effect of negatively charged DS [26]. In the next stage, the addition of PEI increased the size of APENs (313.8 ± 5.2 nm and 0.32 ± 0.01) and decreased the hydrodynamic diameter of WPENs (249.1 ± 0.8 nm and 0.27 ± 0.00) significantly, at a PEI concentration of 1.5 mg/mL (Figure 1). This event can be explained by the presence of electrostatic interactions among the layers, which led to the possible π−π stacking of the aromatic side chains [24], as well as hydrophobic interactions, and then the formation of more compact structures in WPENs. 

DLS data also demonstrated a significant difference between the hydrodynamic diameter of PENs and RIF/PENs (*p* < 0.05) with acceptable PDI values (Table 1). The more compact structure of PENs, especially in the presence of RIF, should be attributed to the hydrophobic interactions among RIF and hydrophobic domains of CS derivatives. The hydrophobic portions try to locate in the interior portion of PENs by clustering together and reducing their contact with the hydrophilic environment [27]. As shown in Table 1, in spite of variations in PENs’ diameters analyzed by SEM, TEM, and DLS, these techniques confirmed that the mean diameter of free APENs was significantly larger than other particles (*p* < 0.05). According to Eaton et al., the above techniques measure particle size by using different approaches, and, as such, the reported size estimates are different, as expected [28]. 

**Figure 2:**
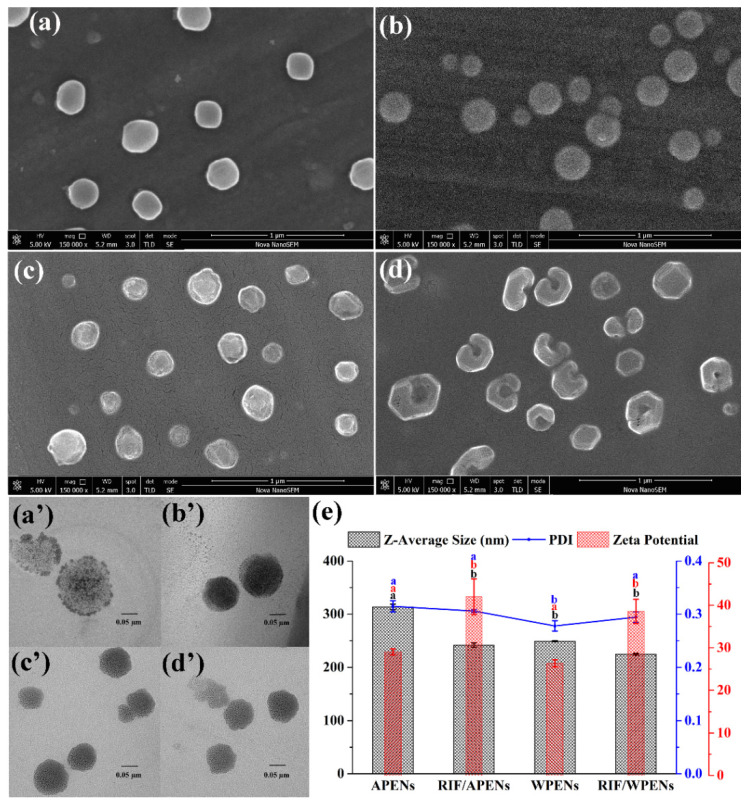
Evaluation of size, morphology, ζ-potential and PDI of PENs by different techniques which exhibit SEM micrographs of APENs (**a**); RIF/APENs (**b**); WPENs (**c**); and RIF/WPENs (**d**). TEM images of APENs (**a’**); RIF/APENs (**b’**); WPENs (**c’**); and RIF/WPENs (**d’**); and DLS analyses (**e**).

SEM micrographs indicated regular size distribution and spherical shape of PENs with no aggregation (Figure 2a–c), which was a little different in terms of RIF/WPENs by the appearance of concave deformations in some of the nanoparticles, as well as an invagination or an inner hole in others (Figure 2d). This unexpected observation can be explained by the presence of a few large, bulky aromatic side chains of W on the surface, which tend to be inside after hydrophobic interactions with hydrophobic cargo. TEM images exhibited a dark ring structure around a large loose core of APENs (Figure 2a’), as opposed to the compact structure of WPENs (Figure 2c’), which changed to more compact structures with no significant difference between two PENs in the presence of RIF (Figure 2b’,d’). It is supposed that, in the absence of RIF, there are weaker hydrophobic interactions among the small aliphatic side chain of ACS than the large rigid indole side chains of WCS, which is also confirmed by ζ-potential values. According to (Figure 2e) and Table 1, there was no considerable difference between ζ-potential of APENs (29.0 ± 0.7) and WPENs (26.3 ± 0.8), which increased significantly in the presence of RIF to 42.0 ± 4.2 (*p* = 0.006) and 38.5 ± 2.8 (*p* = 0.002), respectively. The positive ζ-potential is associated with positively charged amino groups on the surface of particles, whose formation of hydrophobic interactions among RIF and the amphiphilic core leads to more amino groups being exposed to the environment and increases the ζ-potential. Moreover, in addition to ionic interactions among the layers, the intrinsic difference of the amino acids’ structures significantly affects the size and morphology of the particles; however, the substitution effect should be far lower than other factors due to the same approximate quantity.

### 2.2. Evaluation of Colloidal Stability of PENs

In this study, the colloidal stability of the PENs was evaluated by DLS in distilled water and PBS (10 mM, pH 7.4) upon storage at 4 °C for two weeks. According to Table 2, the size variations of PENs were negligible, and they presented no aggregates confirmed by PDI values. Z-average size of APENs changed from 302.1 ± 5.2 to 304.2 ± 7.4, and WPENs from 233.5 ± 9.4 to 249.5 ± 5.2, in distilled water over two weeks. The hydrodynamic diameter of APENs (294.1 ± 10.4) and WPENs (239.1 ± 3.5) stored in PBS 10mM exhibited no significant change. Considering these features, the particles demonstrated colloidal stability in the presence and absence of salt for two weeks, which can be attributed to inter/intramolecular interactions. It was expected that salt ions lead to increasing size by neutralization of electrostatic interactions and the passive osmotic diffusion of H_2_O [29]. Nonetheless, alkaline PENs using deprotonation of amino groups, lowering electrostatic attractions, and partial hydrogen bonding displacement can improve the stability of PENs [26]. Besides, the stability of the particles can be attributed to the hydrophobic interactions among hydrophobic moieties of the CS derivatives [24], as well as the small size and narrow particle size distribution [30].

### 2.3. HNMR, Elemental Analysis and Infrared Spectrophotometry Analysis

**Figure 3:**
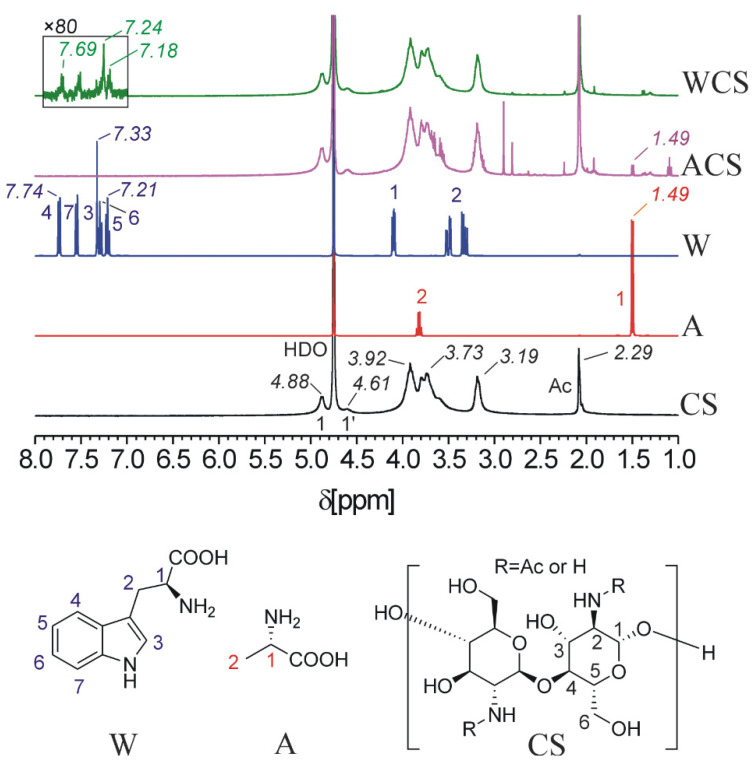
^1^H-NMR spectra of CS, A, W, ACS and WCS (303 K, 1% CD_3_COOD in D_2_O). Signal assignment is given using color-coded numbers. Positions at acetylated unit of CS are denoted by an apostrophe.

Figure 3 shows ^1^H NMR spectra of CS, A, W and modified CS in CD_3_COOD/D_2_O to characterize the CS derivatives. The proton assignment of CS is as follows: 2.29 ppm (CH_3_ group of *N*-acetyl glucosamine); 3.19 ppm (H(2) proton of the glucosamine and *N*-acetyl glucosamine); 3.73–3.92 ppm (ring protons of H(3), H(4), H(5) and H(6) of the glucosamine and *N*-acetyl glucosamine); 4.61 (anomeric H(1) of *N*-acetyl glucosamine); and 4.88 (anomeric H(1) of glucosamine) [31]. The signal of the A methyl group 1.49 ppm (labeled as 1) is observed in ACS spectra, but the signal of the α-CH group (labeled as 2) has been overlapped by the strong signals of the CS backbone. In W molecule, the signals were assigned according to the literature [32]. In the spectrum of WCS, the two aliphatic signals of W residue have been overlapped completely by the strong signals of the CS backbone. However, the signals of the aromatic part of the W residue can be clearly observed in the spectrum, as indicated by an enlarged view in the inserted box in Figure 3. Therefore, the successful grafting of the amino acids onto CS is confirmed by the clear signals of amino acid residues in the spectra of ACS and WCS. We estimated the degree of substitution of ACS (1.7%) by the integral values of the A residue signal H(1) and signals of the CS anomeric H-atoms, and WCS (0.9%) by integral intensity of aromatic H-atoms of W-residue. The substitution degrees of A and W grafted onto CS were also determined by elemental analysis (C, N, H), which demonstrated no significant difference between the substitution degrees of A (5.93 ± 0.44%) and W (6.55 ± 0.88%) grafted onto CS. The difference between these two techniques can be owed to certain limitations, such as incomplete solubilization of the samples in the deuterated solvent, which led to an inaccurate analysis of modified polymers with low substitution degrees [33]. 

**Figure 4:**
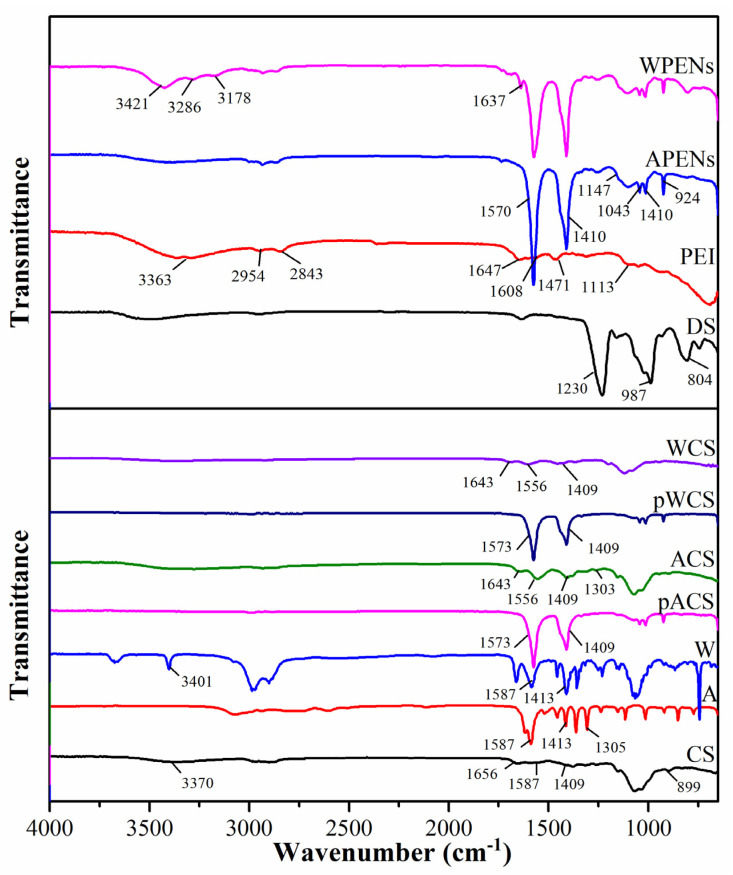
FTIR spectra of neat materials (CS, A, W, DS, PEI) physically (pACS and pWCS) and chemically (ACS, WCS), a mixture of amino acids with CS, and nanostructures (APENs and WPENs).

An FT-IR technique was also performed to determine the chemical composition, functional groups, and possible interactions of the structures. Figure 4 demonstrated FT-IR spectra of pure materials and the physical/chemical mixture of hydrophobic amino acids with CS and PENs. FT-IR spectra of pure CS show characteristic peaks assigned to 899 cm^−1^ (pyranose ring), 3370 cm^−1^ (OH and NH_2_ stretching), 1656 cm^−1^ (amide I), 1587 cm^−1^ (amide II), and 1409 cm^−1^ (CH_3_ bending vibrations) [24]. In the physical mixtures (pACS and pWCS), there are two sharp peaks at 1409 cm^−1^ and 1573 cm^−1^ assigned to symmetric CH_3_ bending and N–H bending of amine, respectively, which are blue shifted to 1413 cm^−1^ and 1587 cm^−1^ in A and W [24]. In the chemical mixture of ACS and WCS, the intensity of symmetric CH_3_ bending reduced, N–H bending of amine shifted to 1556 cm^−1^, and carboxamide I band appeared at about 1643 cm^−1^. Therefore, the existence of CH_3_ bending absorbance peak at 1303 cm^−1^ in ACS, shifting of the sharp peak of N-H stretching vibration of the indole ring at 3401 cm^−1^ to a much-broadened peak at 3423 cm^−1^ in WCS, and appearance and shifting of the absorbance peaks of carboxamide I and II bands at about 1643 and 1556 cm^−1^, were indicative of successful graft of A and W onto CS [24]. As shown in Figure 4, a pure DS spectrum demonstrated several identical absorption bands, including asymmetric and symmetric SOO¯ stretching vibrations at ~1230 cm^−1^ and 987 cm^−1^, confirmed the presence of a sulfate-group in DS spectrum, as well as the bands at about 804 cm^−1^ derived from asymmetric S–O–S vibration [34]. An FT-IR spectrum of PEI exhibited the characteristic absorption peaks of amine groups (3363 cm^−1^ and 1647 cm^−1^), C–N stretching (1113 cm^−1^), C–H deformation (1471 cm^−1^), N–H deformation (1608 cm^−1^), and the stretching vibration of C-H bonds of the alkyl chain (2954 and 2843 cm^−1^) [26]. The spectra analysis of the APENs and WPENs showed the peaks of sulphones (1147 cm^−1^), the sulfo-group (924 cm^−1^, 1014 cm^−1^ and 1043 cm^−1^) and weak N–H stretching vibrations (648 cm^−1^) [35]. High intensity of a few corresponding sharp bands, including 1570 cm^−1^ (N–H bending of amine) and 1410 cm^−1^ (symmetric CH_3_ bending), was also observed, which can be attributed to polyplex formation via great electrostatic interaction. In WPENs, a few sharp peaks near to 3421 cm^−1^ (primary amines), 3286 cm^−1^ and 3178 cm^−1^ (O-H (H-bonded)), and 1637 cm^−1^ (C=O (amide I band)) cm^−1^ are also exhibited, which can be related to a larger number of functional groups involved in hydrogenic and/or ionic interactions. Overall, these characteristic absorbance peaks confirm that the particles have been achieved successfully.

### 2.4. XRD Analysis

**Figure 5:**
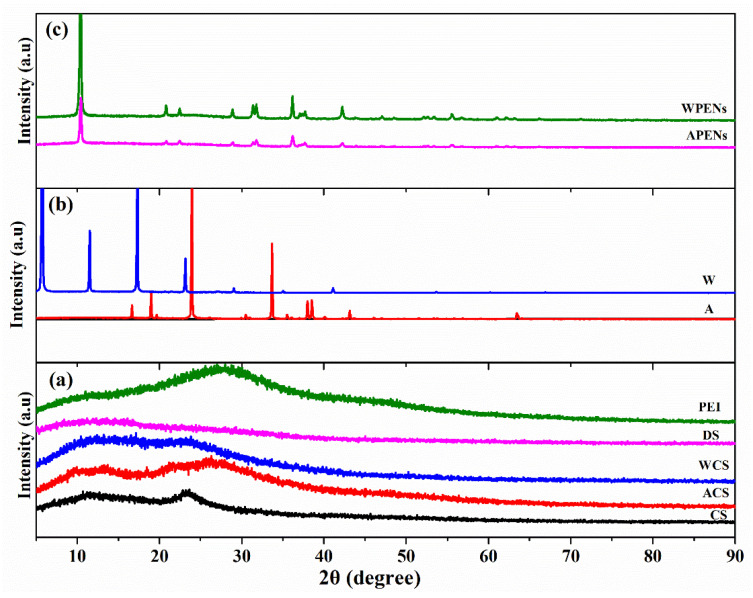
X-ray diffractograms of CS, ACS, WCS, DS, and PEI (**a**); A, W (**b**); and PENs (APENs and WPENs) (**c**).

The nature and crystalline structure of neat CS, DS, PEI, A, W, then, the CS derivatives (ACS, WCS) and the nanoparticles (APENs and WPENs), were identified by an XRD technique. According to Figure 5, the X-ray diffraction pattern of PEI demonstrated almost broad diffraction at 27.4° [36], while DS had one broad peak at 12.8° [37], and CS showed two strong peaks at 11.5° and 23.7° [25], which are characteristics of a polymer with partial crystallinity [38]. The diffractograms of ACS and WCS compared with CS exhibited some changes in diffraction angles, peak intensity, and peak width. The crystalline index (CrI) value of ACS and WCS also increased to 42.67% and 44.48%, respectively, in comparison with CS (40.03%). Therefore, A with CrI value of 77.09% and the appearance of the peaks around 16°, 19° and 23° [39], and W with CrI value of 81.48% and the appearance of the peaks around 5°, 11°, 17° and 23° [40], affected the CS diffractogram and increased crystallite size. Finally, the appearance of several strong peaks in the diffractogram of PENs confirmed the high degree of crystallinity, and the presence of materials affected the crystallinity of the packed structure. The CrI value of the APENs (72.76%) and WPENs (72.66%) confirmed that the PENs transited to a higher crystalline state through the intra/intermolecular interactions. This can be attributed to the hydrophobic bonds among the hydrophobic moieties of the CS derivatives, as well as the electrostatic interactions and hydrogen-bonding among different layers, which intensify intra/intermolecular forces and lead to an increase in crystallinity [41,42].

### 2.5. Calorimetric Analysis 

**Figure 6:**
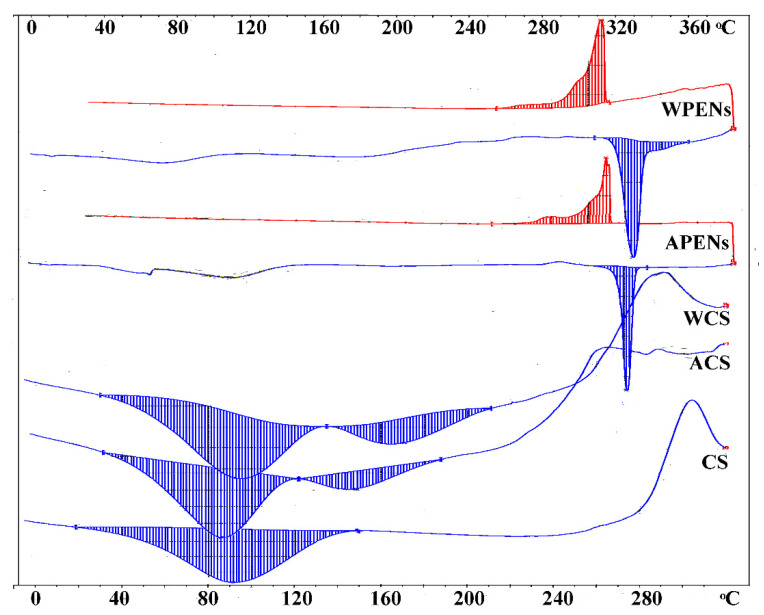
DSC curves of CS, CS derivatives (i.e., ACS, WCS), and PENs (i.e., APENs and WPENs)

A DSC analysis was performed to elucidate the physical state and thermal behavior of the two PENs. According to Figure 6, CS shows a typical polysaccharide thermal trait characterized by an endothermic peak at 91.28 °C, corresponding to polymeric dehydration, and an exothermic peak at 304 °C, corresponding to polymeric degradation. ACS (85.58 °C and 148.61 °C) and WCS (92.43 °C and 170.75 °C) demonstrate phase transformations during the melting procedure [43], which can be discussed by vaporization of water molecules adsorbed on the surface and entrapped in the amphiphilic moieties of CS derivatives, respectively. The DSC of PENs, obtained from (−10) to (+380) °C, indicates the melting and crystallization peaks. It is shown that the melting (endothermal peak) of APENs and WPENs takes place at 321.07 °C and 324.82 °C, and the crystallization (exothermal peak) at 309 °C and 307 °C, respectively. The shifting of melting points towards higher temperatures can be accounted for by changes in solid-state structure of PENs, and confirms the existence of strongly bound water molecules among the different polymeric layers, which requires more energy to be removed [44]. The other important event of these calorimetric curves is the exothermic peak during the cooling phase. This type of crystallization occurs at temperatures well below the melting region, and, commonly, it is observed as a fully irreversible phenomenon due to high nucleation density [45].

### 2.6. In Vitro Release Studies

For illustrating the effect of structural parameters (i.e., amphiphilic core and polycationic shell) and environmental conditions (i.e., pH and antioxidant) on the RIF release process, the encapsulation and release behavior of RIF-loaded PENs were evaluated. The results confirmed that there was no significant difference (*p* > 0.05) between the EE (%) of APENs (88.5 ± 17.1) and WPENs (89.4 ± 15.8), and, also, the LC (%) of APENs (4.0 ± 0.6) and WPENs (4.1 ± 0.5). Therefore, the two different nanoconstructions showed high and approximately equal amounts of EE attributed to the strong hydrophobic interactions of RIF to hydrophobic moieties of the amphiphilic core, and a very low amount of LC (%), discussed by the strong dependency of LC on the weight ratio of nanoparticles, in accordance with Equation (3).

**Figure 7:**
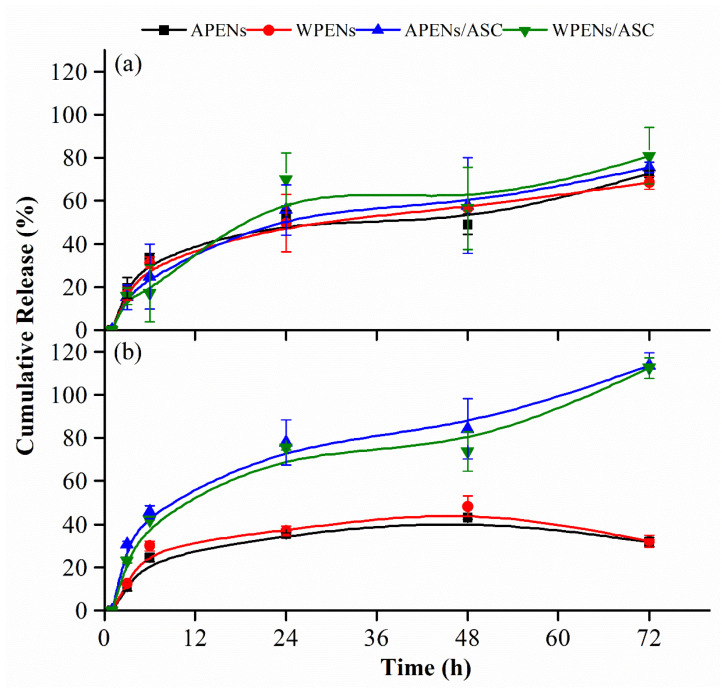
In vitro release profiles of RIF-loaded PENs in different release media at pH values of 7.4 (**a**) and 8.5 (**b**) in the presence and absence of ASC.

Due to the alkaline environment of chronic nonhealing wounds, which have lower healing rates compared with neutral or acidic wounds [46] and high stability of the alkaline PENs [26], the in vitro release profiles of RIF-loaded PENs were evaluated at alkaline (8.5) and physiological (7.4) pHs. As shown in Figure 7, the in vitro release profiles of RIF-loaded PENs were assigned to a biphasic process, including an initial burst release for about 6 h and a subsequent gradual release for 72 h. The initial burst and diffusion of adsorbed RIF into the media were the result of the high swelling and dissolution rate of the shells. The subsequent slow release was associated with the hydrophobic interaction of RIF to hydrophobic moieties of amphiphilic core, and, most likely, from peeling off and dissolution of the remaining multilayer structure of PENs [26].

According to Figure 7, both PENs demonstrated the same in vitro release profiles. Table 3 also confirmed that there was no statistical difference between the cumulative release of APENs and WPENs at two different pH values, and in the presence and absence of ASC. Therefore, in spite of structural differences between two species of hydrophobic moieties, they played the same role in RIF releasing behavior. Figure 7a indicated that the presence of ASC at pH values of 7.4 did not influence RIF release behavior, but Figure 7b clearly showed the effective role of ASC in alkaline release media. At alkaline pH and an absence of ASC, PENs indicated significantly lower release rates of RIF, as the gradual release of RIF increased to 43.0 ± 0.9 (APENs) and 48.3 ± 4.6 (WPENs) at 48 h, and then decreased to 31.8 ± 2.1 (APENs) and 32.1 ± 2.8 (WPENs) at 72 h. These low amounts of RIF release and the final decline phase can be attributed to poor stability of RIF at alkaline pH. Figure 7b also demonstrated that the gradual release of RIF increased to 84.0 ± 13.9 (APENs) and 73.7 ± 9.1 (WPENs) at 48 h, and was then accompanied by a sharp increase to 113.5 ± 6.0 (APENs) and 112.3 ± 4.7 (WPENs) at 72 h. Therefore, ASC protected RIF at the alkaline pH and caused a gradual increase in the RIF release rate for 48 h. Scolari et al., also declared that ASC significantly decreased the oxidative degradation of RIF after releasing alginate/CS nanoparticles, as the concentration of ASC increased [13]. The sharp release rate at 72 h can be explained by RIF and ASC oxidation, and the interactions among the byproducts in alkaline media, which affected UV absorbance. Moreover, ASC at a pH value of 8.5 led to a significant increase of RIF release (*p* < 0.05) in comparison with the media containing no ASC.

### 2.7. Degradation Studies 

**Figure 8:**
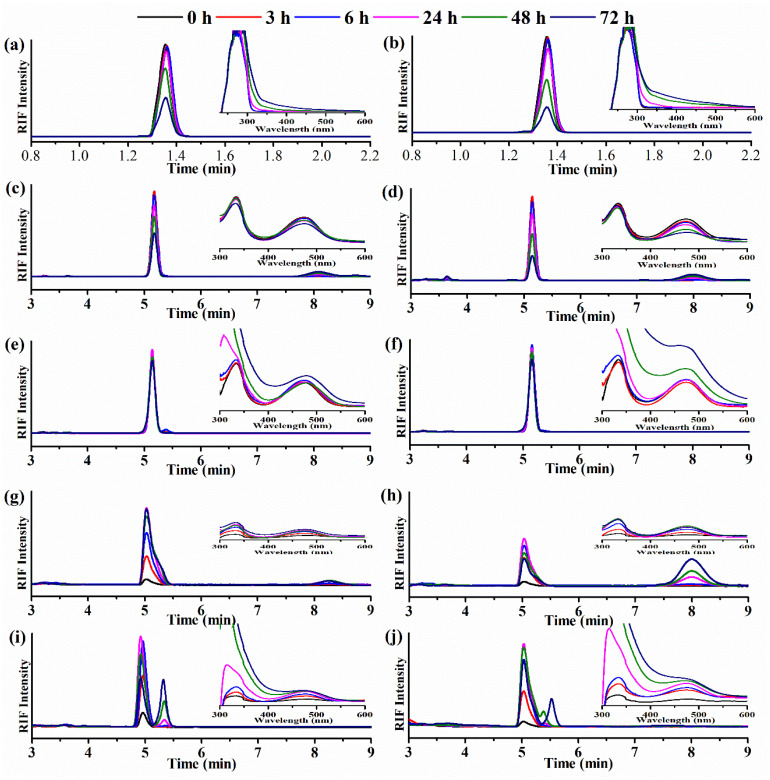
HPLC and UV/Vis spectra of ASC/pH 7.4 (**a**); ASC/pH 8.5 (**b**); RIF/pH 7.4 (**c**); RIF/pH 8.5 (**d**); RIF/ASC/pH 7.4 (**e**); RIF/ASC/pH 8.5 (**f**); RIF/APENs/pH 7.4 (**g**); RIF/APENs/pH 8.5 (**h**); RIF/APENs/ASC/pH7.4 (**i**) and RIF/APENs/ASC/pH 8.5 (**j**).

Owing to the similar release rate of the two PENs, degradation studies were continued by APENs. Herein, for illustrating the protective effect of antioxidants and PENs on RIF, the degradation rate of RIF was evaluated at different release media (i.e., physiological and alkaline pHs) at 37 °C by UV/Vis spectrophotometry and HPLC. Previous studies referred that ASC, as an antioxidant, decreased RIF oxidation [47]. Therefore, the degradation rate of ASC and its exponential effect on RIF were evaluated at different release media. As shown in Figure 8a,b, the UV spectrum of ASC at two different pHs showed one maximum at 276 nm, which became broader and shifted toward the red region (bathochromic effect), particularly after 24 h. It is in agreement with Maniyar, et al., who clearly indicated an altered chemical behavior of ASC at different pH values [48]. These changes are more obvious at alkaline pH due to the greater oxidation of ASC (Figure 8b). HPLC analysis also confirmed these data by starting peak area reduction after 24 h, at a retention time of 1.35 min, which was greater at pH values of 8.5 (Figure 8a,b). According to Table 4, ASC degradation started at t = 0 h and increased gradually for 72 h. The amount of degradation at alkaline pH was significantly higher than physiological pH after 24h (*p* = 0.013), and increased to 58.3 ± 0.0% at pH 7.4 after 72 h, in comparison to 72.2 ± 0.11% at pH 8.5 (*p* = 0.000). 

RIF is a labile drug whose degradation is influenced by temperature, length of storage, and pH [47]. Figure 8c,d shows the spectrophotometric and chromatographic behavior of RIF in different environments, and Table 4 lists the percentage of RIF degradation. The spectrum of RIF under physiological and alkaline pHs showed two stabilized maxima at 334 and 475 nm for 6 h, accompanied by a decreasing and shifting of absorbance peaks until 24 h. These changes can be attributed to starting strong degradation from 24 h. HPLC data also confirmed that, after 24 h, RIF began to degrade strongly by reducing the peak area at a retention time of 5.15 min, and the appearance of a new peak, at approximately 7.99 to 8.09 min, at two pHs. The new peak was suspected to be RIFQ, as RIF transforms into RIFQ as a major degradation product of alkaline degradation in the presence of atmospheric oxygen and room temperature. It seemed that autoxidation of RIF led to the conversion of the naphthyl core into naphthoquinone with distinctive biochemical properties [49]. Therefore, the RIF peak area reduction, and the degradation substance peak appearance and heightening, were associated with increasing RIF degradation over time. 

According to Table 4, the extent of degradation at pH values of 8.5 (7.0 ± 0.2) was significantly greater than at a pH value of 7.4 (5.1 ± 0.1) from 6 h (*p* = 0.001), which increased to 70.4 ± 0.1 and 50.6 ± 0.4 at pH values of 8.5 and 7.4 (*p* = 0.000), respectively. According to Alves et al., RIF stability varies by pH due to the amphoteric nature, and the highest stability is attributed to near-neutral solutions. They also confirmed that the addition of ASC to the solutions increases the solubility of RIF and decreases its oxidation [50].

This assay was continued by RIF in the release media containing ASC. At pH 7.4, the spectra of RIF were unchanged during the first 6 h, but a significant rise (334 nm) and redshift (475 nm) were detected at 24–48 h, and continued by the significant growth of these two absorbance peaks at 72 h (Figure 8e). At pH 8.5, the spectra of RIF followed the same model of pH 7.4 for 24 h, and then a very high–slope curve at 334 nm and an upward trend in the absorbance peak of 475 nm started from 48 h (Figure 8f). It might be characterized by the formation of another byproduct which affected the absorbance peaks of 334 nm and 475 nm strongly. Levy et al., evaluated the effect of ASC on anthocyanins’ stability by spectral techniques (i.e., UV/Vis spectrophotometry and HPLC), and declared that the direct condensation of ASC with anthocyanins, or the formation of hydrogen peroxide and oxidative cleavage, might lead to significantly enhanced degradation [51]. However, HPLC analysis demonstrated that RIF was more stable in the presence of ASC due to the disappearance of the degradation substance peak, as well as the RIF peak area reducing at 48 h at the retention time of 5.16. Table 4 also confirmed that ASC showed a significant protective effect against RIF degradation at two pH values, with considerable impact on pH 7.4 (*p* < 0.05). RIF degradation started with the rate of 0.1 ± 0.0 at 3h and continued to 12.6 ± 0.1 for 72 h at pH 8.5, and started at 48 h with the rate of 3.1 ± 0.3 and continued to 9.2 ± 0.4 for 72 h at pH 7.4. 

Due to the high efficiency of nanoparticles in protecting labile drugs, RIF degradation was compared after PENs’ entrapment at physiological and alkaline pHs. According to Figure 8h, the UV/Vis data show a gradual release of RIF by an increased peak height at both wavelengths (334 nm and 475 nm) for 24 h, and then a constant release rate at 48 h and, finally, an increasing (pH 7.4) and decreasing (pH 8.5) spectral peak height at 72 h at both wavelengths. Decreasing the spectral peak height at 72 h can be attributed to RIF degradation at pH 8.5. This is confirmed by the HPLC data by decreasing and increasing the RIF peak area at 48 and 72 h, respectively, as well as the appearance of another peak at a retention time of 8.25 min from 6 h, which kept increasing for 24 h at pH 7.4. Whereas, at pH 8.5, the RIF peak area began to decrease strongly between 48–72 h, and the degradation substance peak at a retention time of 8.01 min appeared after 6 h, which continued to increase for 72 h. According to Table 4, PENs were able to protect RIF efficiently at two pHs, owing to the significant difference (*p* < 0.05) between the degradation rate of free RIF and RIF released from PENs. However, at pH 7.4, the protective effect of PENs was considerably greater than pH 8.5 (*p* < 0.05), as there was no significant degradation during storage time. Nonetheless, at pH 8.5, RIF degradation was 5.4 ± 0.5 for 24 h and then increased sharply to 42.4 ± 1.7 at 72 h. Therefore, it can be concluded that PENs could protect RIF against harsh environments, but not the same as physiological pH. 

According to Table S1, PENs were able to protect RIF efficiently at two pHs, owing to the significant difference (*p* < 0.05) observed between the degradation rate of free RIF and RIF released from PENs. However, at pH 7.4, the protective effect of PENs was considerably greater than pH 8.5 (*p* < 0.05), as there was no significant degradation during storage time. Nonetheless, at pH 8.5, RIF degradation was 5.4 ± 0.5 for 24 h and then increased sharply to 42.4 ± 1.7 at 72 h. Therefore, it can be concluded that PENs could protect RIF against harsh environments, but not the same as physiological pH.

Finally, the simultaneous protective effect of PENs and ASC on RIF degradation was evaluated at physiological and alkaline pHs. Figure 8i,j showed that the released RIF followed the similar spectral profiles at two different pH values of 7.4 and 8.5. The spectra of RIF at 334 nm increased dramatically from 24 h, which confirmed that this absorbing wavelength was generally not applicable for the analysis of highly concentrated ASC solutions. Nonetheless, the spectra of 475 nm climbed gradually for 24 h, then stabilized until 48 h, and, finally, increased sharply at 72 h. The last rapid increase in the spectrum can be attributed to high RIF and ASC degradation, and then byproduct formation. HPLC analysis shows, in Figure 8i,j, an increase in a concentration expressed as the total peak area of RIF for 24 h, and then a decline for 72 h at both pHs. At pH 7.4, a blue shift of RIF peak (4.91 to 5.04 min) and elution of a new peak, at a retention time of 5.35 and 5.62 min from 6h with increasing intensity for 72 h, were also observed. At pH 8.5, peak splitting occurred from 24 h, and it was completely separated at 72 h. Table 4 also demonstrated starting RIF degradation from 24 h at two different pHs, which then increased to 44.6 ± 4.6 at pH 7.4 and 21.6 ± 3.0 at pH 8.5 at 72 h. It was expected that RIF degradation was decreased by the synergistic effect of PENs and ASC at two different pHs, due to separate protective effects of ASC and PENs. Nonetheless, the comparison among the spectra, the peak areas, and the degradation rates confirm that the possible synergistic effect was observed only at alkaline pH. Moreover, in spite of slowing down RIF degradation in the presence of ASC/PENs, it can be declared that the presence of ASC at alkaline pH is necessary, as opposed to physiological pH.

## 3. Materials and Methods

### 3.1. Materials 

Low molecular weight CS (MW of 50–190 kDa and degree of deacetylation ≥75%), DS sodium salt (MW of 7–20 kDa), PEI (average MW 1.3 kDa), polysorbate 80, A, W, RIF, ASC, *N*-(3-Dimethylaminopropyl)-*N*′-ethylcarbodiimide (EDC), *N*-Hydroxysuccinimide (NHS), acetic acid (glacial, ≥99.85%), sodium chloride, disodium hydrogen phosphate, potassium chloride, potassium dihydrogen phosphate, methanol, and cellulose dialysis tubing, with cut off 12 kD MWCO, were purchased from Sigma-Aldrich (St. Louis, MO, USA). All chemicals used in the study were of analytical grade.

### 3.2. Synthesizing and Characterization of CS Derivatives 

Amino acids (A and W) grafted onto CS were synthesized using EDC/NHS in stoichiometric amounts, as previously reported [24]. The amounts of amino acids and the equal amounts of EDC and NHS were 0.2 equivalent/[NH_2_] of CS and 1.5 equivalent/[COOH] of amino acids, respectively. In brief, EDC/NHS was added to the dissolved amino acids under uniform stirring at 4 °C for 30 min. Thereafter, the mixture was gradually added into the CS solution, dissolved in acetic acid (2% *v*/*v*) with pH 5.0 dropwise under constant stirring at 4 °C for 30 min, and continued for 24 h at room temperature. After dialysis for 3 days against double deionized water, the excess coupling reagents and unreacted amino acids were eliminated. Finally, the products were freeze-dried and stored at −20 °C for further assays, including FT-IR, elemental analysis, ^1^H-NMR, XRD and DSC. FT-IR spectra were carried out with a Nicolet iS5 spectrometer (Thermo Fisher Scientific, Madison, WI, USA) at 64 scans and a resolution of 4 cm−1 over a wavenumber range of 4000–400 cm^−1^. Elemental analysis was also performed by an Organic Elemental Analyzer (FLASH 2000 CHNS/O + MAS200R, Thermo Fisher Scientific, Sunnyvale, CA USA). ^1^H-NMR was conducted using a JEOL NMR spectrometer (400 MHz, MA, USA) equipped with a 5 mm ROYAL HFX Probe. ^1^H NMR spectra were recorded at 303 K in 1% CD_3_COOD (99.5% D) in D_2_O (99.8% D) as solvent, with 64 scans and 2 dummy scans. Spectra were referenced to the signal of residual HDO (δ 4.75 ppm). Thermal analysis of the ingredients was determined by DSC (Mettler Toledo, Greifensee, Switzerland) at a heating rate of 10 °C/min over a temperature range of (−10) to (+330) °C for raw materials, and −10 to +380 °C for the PENs under a nitrogen purge set to 50 mL/min [52]. The powder XRD patterns were also recorded on a Rigaku MiniFlex 600 diffractometer (Tokyo, Japan) equipped with a CoKα (λ = 1.7903 Å) X-ray tube (40 kV, 15 mA), with the Bragg angle ranging from 5° to 90°. Then, CrI was obtained from the ratio of the area of the crystalline contribution (A_cryst_) to the total area of the diffractogram (A_total_), as proposed by Osorio-Madrazo et al. [53].

CrI (%) = 100 × A_cryst_/A_total_ (1)

### 3.3. Synthesizing and Characterization of PENs 

For the preparation of PENs, CS derivatives were dissolved in 1% (*w*/*v*) acetic acid solution (500 rpm, 24 h, RT), adjusted pH to 5.0, and then mixed by 0.5% polysorbate 80 (500 rpm, 60 min, 45–50 °C). To optimize the formulation, different volumes of alkaline DS (1 mg/mL, pH 8) were added to the CS solution under stirring (500 rpm, 10 min) to obtain the smallest z-average size of the core. Thereafter, an optimized PEI concentration was determined by mixing different alkaline PEI volumes (10 mg/mL, pH 8) with the core solution under stirring (500 rpm, 10 min). The final optimized formulation contained 1.1 mL DS (1.0 mg/mL, pH 8.0), 0.45 mL PEI (10 mg/mL, pH 8.0), and 6 mL of the CS solution (1.0 mg/mL, pH 5.0). For the preparation of RIF-loaded PENs, 0.6 mL RIF solution in DMSO/distilled water (1.0 mg/mL), due to the acceptable stability of RIF in an organic solvent such as DMSO [1,54], was added to the CS derivatives’ solution under stirring (500 rpm, 15 min, 4 °C and in a dark glass bottle). Hereafter, the abbreviations of APENs and WPENs were used for the PENs synthesized by ACS and WCS, respectively. Then, the particle size, ζ-potential, and morphology of PENs were determined at 25 °C using DLS (model 3600, Malvern Instruments Ltd., Worcestershire, UK), SEM (Nova 450 NanoSEM, FEI, Czech Republic) and TEM (JEM 2100, JEOL Ltd., Tokyo, Japan) operated at 5.00 kV and 160 kV accelerating voltage, respectively. For SEM, the dried suspension on a piece of aluminum foil was placed on the SEM specimen stub with a double-sided carbon adhesive disc (Taab, Berkshire, UK), and sputter-coated with gold/palladium (SC7620 Mini Sputter Coater, Quorum Technologies, Laughton, UK, 10 mA for 45 s), and TEM sample preparation was done by a dried suspension on a carbon-coated 300-mesh copper grid (Structure Probe Inc., West Chester, PA, USA). Colloidal stability of the PENs was also evaluated in distilled water and PBS (10 mM, pH 7.4) at 4 °C by DLS at predetermined time intervals for two weeks. Freeze-dried samples were used for further assays, including FT-IR, XRD, and DSC. 

### 3.4. Drug Loading and in Vitro Release Assays of PENs

Drug loading and in vitro release of RIF-loaded PENs were evaluated using the dialysis technique and in the presence of PBS. For evaluation of encapsulation efficiency (EE) and loading capacity (LC) of RIF-loaded PENs, the dispersions were placed into 100 mL PBS 10 mM, pH 7.4 by a dialysis tube, and, then, the entire system was kept in an orbital incubator (Stuart SI500, ST15 OSA, UK) at 37 ± 0.5 °C, 40 rpm for 1 h. EE (%) and LC (%) were calculated according to Equations (2) and (3), respectively [55], where Total RIF was the amount of primary RIF added to the solution and Free RIF was evaluated by UV/Vis spectrophotometry (CARY 300 Conc, Agilent, Victoria, Australia) at 475 nm versus a calibration curve (R2 = 0.9994, y = 0.0261c − 0.0173).

EE (%) = ((Total RIF − Free RIF)/Total RIF) × 100(2)

LC (%) = ((Total RIF − Free RIF)/Total Weight of PENs) × 100(3)

Afterward, for evaluation of in vitro release rate, the medium was replaced with 50 mL PBS 10 mM (pH 7.4 and 8.5) in the presence and absence of ASC (1.0 mg/mL), and the entire system was kept in an orbital incubator at 37 ± 0.5 °C, 40 rpm. At predetermined time intervals, 3 mL of the medium was drawn and replaced with a fresh medium, keeping the volume constant. The amount of RIF released into the media was evaluated by the above-described UV/Vis spectrophotometry method.

### 3.5. RIF Degradation Assays

To evaluate the protective effect of PENs and ASC on RIF, RIF degradation was analyzed in the in vitro release media in the presence and absence of PENs and ASC at different pHs (7.4 and 8.5), and a constant temperature of 37 ± 0.5 °C. Therefore, the dark glass bottles containing (1) ASC (1.0 mg/mL), (2) RIF (1 µg/mL), (3) RIF/ASC, (4) dialysis tubes containing RIF-loaded PENs, and (5) RIF-loaded PENs/ASC were placed into 50 mL of PBS 10 mM (pH 7.4 and 8.5), and, then, the entire system was kept in an orbital incubator (Stuart SI500, ST15 OSA, UK) at 37 ± 0.5 °C, 40 rpm for 72 h. At predetermined time intervals, 3 mL of the media were drawn and quantified by UV/Vis spectrophotometry and an HPLC Dionex UltiMate 3000 Series (Thermo Fisher Scientific, Sunnyvale, CA, USA).

HPLC is a sensitive and specific method for the assay of drugs and their metabolites, which has been utilized for the confirmation of UV/Vis spectrophotometry analysis. The separation was performed on a reversed-phase column Kinetex 2.6u C18 100 A (150 mm × 4.6 mm; Phenomenex, Torrance, CA, USA) equipped with a security guard column (Phenomenex, Torrance, CA, USA) at 40 °C. A mixture of HPLC grade Methanol and 0.01 M sodium phosphate buffer (pH 5.2) was used as a mobile phase (61:39, *v*/*v*) at a flow rate of 1.00 mL/min and a total isocratic run of 9 min. The sampler was set to 5 °C, and volumes of 20 µL (standard; samples of RIF and RIF-loaded PENs) and 5 µL (ASC sample) were injected onto the column. Eluted ASC and RIF were detected using wavelengths of 245, 274, 334 and 475 nm. RIF quantification was performed by an external calibration method, and the calibration curve’s equations were as follows: y = 0.7296c − 0.7707 (R^2^ = 0.9995), y = 0.4641c − 0.4886 (R^2^ = 0.9995), y = 0.5644c − 0.5922 (R^2^ = 0.9995), and y = 0.3376c − 0.355 (R^2^ = 0.9994), for wavelengths of 245, 274, 334, and 475 nm, respectively. The concentrations of RIF were calculated from the results of 245 nm.

### 3.6. Statistical Analysis 

All experiments were done in triplicate, and the data were presented as mean ± standard deviation. Statistical analysis was carried out using Microsoft Excel 2010 (SRC, CA, USA) and IBM SPSS Statistics 26 (Armonk, NY, USA). One-way and two-way analysis of variance (ANOVA) and independent samples of Student’s *t*-tests were used to compare the mean data using an alfa error of 0.05.

## 4. Conclusions

This study was initiated to circumvent the main causes of poor results obtained during RIF assays, and subject them to the harsh environment of chronic alkaline wounds. Our hypotheses were that amphiphilic chitosan core provided remarkable entrapment and protection of RIF, alkaline shell induced high stability of PENs at alkaline pH, and ASC protected RIF in the release media against alkaline pH. The results led us to substantially different conclusions, including (1) the different amphiphilic core structure only affected the morphology of the two PENs, but not the other physicochemical properties and release profiles; (2) ASC significantly improved release rates of RIF at alkaline pH, in comparison to physiological pH; (3) ASC alone considerably decreased degradation rates of RIF at two different pHs; (4) the alkaline PENs and ASC showed a synergistic protective effect on RIF degradation at pH 8.5, as opposed to pH 7.4; and (5) UV/Vis spectrophotometry was an inapplicable technique for the evaluation of RIF degradation in the presence of ASC. Overall, the controlled drug delivery system showed the capacity to protect RIF against the actual pH in chronic wounds, guarantee bioavailability, and provide much more effective treatment, especially in the presence of ASC.

## Figures and Tables

**Table 1:** Evaluation of the size (nm), PDI, and ζ-Potential of PENs and RIF-loaded PENs.

		APENs	RIF/APENs	WPENs	RIF/WPENs
SEM	Size	296.7 ± 28.2 ^a^	224.1 ± 50.9 ^b^	235.0 ± 46.5 ^b^	226.4 ± 50.7 ^b^
TEM	Size	271.9 ± 38.8 ^a^	215.0 ± 30.6 ^b^	220.0 ± 40.4 ^b^	184.9 ± 34.4 ^b^
DLS	size	313.8 ± 5.2 ^a^	241.9 ± 4.0 ^b^	249.1 ± 0.8 ^b^	224.7 ± 1.8 ^c^
	PDI	0.31 ± 0.01 ^a^	0.31 ± 0.00 ^a^	0.28 ± 0.01 ^a^	0.29 ± 0.01 ^a^
	ζ-potential	29.0 ± 0.7 ^a^	42.0 ± 4.2 ^b^	26.3 ± 0.8 ^ac^	38.5 ± 2.8 ^b^

n = 3, Mean ± Standard Deviation, the different letters (a, b, c) in the same row indicate significant differences between the means of size, PDI and ζ-Potential (*p* value < 0.05), and the values marked with the same letters are not statistically different.

**Table 2:** Physicochemical characterization of PENs during two weeks.

	Time (d)	Z-Average Size (nm)	PDI	Z-Average Size (nm)	PDI
	1	302.1 ± 5.2	0.37 ± 0.03	233.5 ± 9.4	0.19 ± 0.02
H_2_O	7	321.8 ± 13.3	0.27 ± 0.00	243.9 ± 2.0	0.25 ± 0.00
	14	304.2 ± 7.4	0.25 ± 0.00	249.5 ± 5.2	0.18 ± 0.01
PBS 10mM	7	311.4 ± 19.4	0.22 ± 0.01	240.0 ± 5.7	0.25 ± 0.00
	14	294.1 ± 10.4	0.18 ± 0.01	239.1 ± 3.5	0.20 ± 0.01

n = 3, Mean ± Standard Deviation, there are no statistically significant differences in the same column among the mean Z-Average size and PDI of PENs.

**Table 3:** The percentage of the cumulative release of PENs after 72 h at two different pH values (7.4 and 8.5) and the presence and absence of ASC.

		A-PENs	W-PENs
pH 7.4	No ASC	72.8 ± 1.1 ^a^	68.3 ± 3.0 ^a^
	ASC	75.5 ± 2.4 ^a^	80.8 ± 13.0 ^a^
pH 8.5	No ASC	31.8 ± 2.1 ^b^	32.1 ± 2.8 ^b^
	ASC	113.5 ± 6.0 ^c^	112.3 ± 4.7 ^c^

n = 3, Mean ± Standard Deviation, the different letters (a, b, c) indicate significant differences between the mean percentage of cumulative release of PENs (*p* value < 0.05), and the values marked with the same letters are not statistically different.

**Table 4:** Percentage of degradation in different environmental conditions and at predetermined time intervals (h).

		0	3		6	24	48	72
ASC	pH 7.4	0.0 ± 0.0		1.1 ± 0.0 ^a^	2.4 ± 0.2 ^a^	8.9 ± 0.2 ^a^	28.8 ± 0.1 ^a^	58.3 ± 0.0 ^a^
	pH 8.5	0.1 ± 0.0		0.1 ± 0.2 ^b^	1.7 ± 0.1 ^b^	14.1 ± 0.1 ^b^	46.8 ± 0.1 ^b^	72.2 ± 0.1 ^b^
RIF	pH 7.4	0.0 ± 0.0		0.0 ± 0.0	5.1 ± 0.1 ^a^	20.7 ± 0.1 ^a^	37.1 ± 0.2 ^a^	50.6 ± 0.4 ^a^
	pH 8.5	0.0 ± 0.0		0.1 ± 0.0	7.0 ± 0.2 ^b^	23.7 ± 0.2 ^b^	50.3 ± 0.4 ^b^	70.4 ± 0.1 ^b^
	ASC, pH 7.4	0.0 ± 0.0		0.0 ± 0.0	0.0 ± 0.0 ^c^	0.0 ± 0.0 ^c^	3.1 ± 0.3 ^c^	9.2 ± 0.4 ^c^
	ASC, pH 8.5	0.0 ± 0.0		0.1 ± 0.0	0.9 ± 0.1 ^d^	5.2 ± 0.1 ^d^	8.8 ± 0.1 ^d^	12.6 ± 0.1 ^d^
RIF/PENs	pH 7.4	0.0 ± 0.0		0.0 ± 0.0	0.0 ± 0.0	4.1 ± 0.2 ^e^	4.1 ± 1.2 ^c^	3.7 ± 0.3 ^e^
	pH 8.5	0.0 ± 0.0		0.0 ± 0.0	0.0 ± 0.0	5.4 ± 0.5 ^f^	20.4 ± 0.2 ^e^	42.4 ± 1.7 ^f^
	ASC, pH 7.4	0.0 ± 0.0		0.0 ± 0.0	0.0 ± 0.0	12.5 ± 1.1 ^b^	24.1 ± 2.7 ^e^	44.6 ± 4.6 ^a^
	ASC, pH 8.5	0.0 ± 0.0		0.0 ± 0.0	0.0 ± 0.0	1.0 ± 0.0 ^g^	3.2 ± 0.1 ^c^	21.6 ± 3.0 ^g^

n = 3, Mean ± Standard Deviation, the different letters (a, b, c, d, e, f, g) in the same column indicate significant differences between the mean percentage of RIF degradation (*p* value < 0.05), and the values marked with the same letters are not statistically different. The degradation of ASC has been evaluated separately.

## Data Availability

Not applicable.

## Supplementary Materials

The following are available online. Table S1. Percentage of degradation at different environmental conditions and predetermined time intervals (h). Figure S1. UV/Vis spectra of ASC/pH 7.4 (a), ASC/pH 8.5 (b), RIF/pH 7.4 (c), RIF/pH 8.5 (d), RIF/ASC/pH 7.4 (e), RIF/ASC/pH 8.5 (f), RIF/APENs/pH 7.4 (g), RIF/APENs/pH 8.5 (h), RIF/APENs/ASC/pH7.4 (i) and RIF/APENs/ASC/pH 8.5 (j).

## Sample Availability

Samples of the compounds are available from the authors on request.
