# Supplementary material for: Nanoparticle-Based Rifampicin Delivery System Development
Source: Molecules. 2021 Apr 3;26(7):2067. doi: 10.3390/molecules26072067 (PMC8038351; doi:10.3390/molecules26072067)
Supplement: Supplementary file 1 [file molecules-26-02067-s001.pdf]

Nanoparticle-Based Rifampicin Delivery System Development

Marjan Motiei [1,*], Luis Pleno de Gouveia [2], Tomáš Šopík [1], Robert Vícha [3], David Škoda [1], Jaroslav Císař [1], Reza Khalili [4], Eva Domincová Bergerová [1], Lukáš Münster [1], Haojie Fei [1], Vladimír Sedlařík [1], Petr Sáha[1]

[1] Centre of Polymer Systems, University Institute, TBU, tr. Tomase Bati 5678, Zlin, Czech Republic, motiei@utb.cz, sopik@utb.cz, dskoda@utb.cz, jcisar@utb.cz, domincova_bergerova@utb.cz, munster@utb.cz, haojie@utb.cz, sedlarik@utb.cz, saha@utb.cz
[2] iMed.UL-Research Institute for Medicines, Faculty of Pharmacy, Universidade de Lisboa, Lisbon, Portugal, lgouveia@campus.ul.pt
[3] Department of Chemistry, Faculty of Technology, TBU, Vavrečkova 275, Zlín, Czech Republic,
rvicha@utb.cz
[4] Department of Paediatrics and Inherited Metabolic Disorders, First Faculty of Medicine, Charles University and General University Hospital in Prague, Ke Karlovu 2, 12808 Prague, Czech Republic, reza.khalili@lf1.cuni.cz
* Correspondence: motiei@utb.cz

Table S1. Percentage of degradation at different environmental conditions and predetermined time intervals (h)

		**0**	**3**	**6**	**24**	**48**	**72**
ASC	pH 7.4	0.08±0.00	1.12±0.01[ʃ]	2.43±0.23[@]	8.99±0.28[£]	28.81±0.11[¥]	58.39±0.06[¢]
	pH 8.5	0.15±0.06	0.19±0.29[ʃ]	1.7±0.13[@]	14.18±0.12[£]	46.86±0.13[¥]	72.24±0.11[¢]
RIF	pH 7.4	0.00±0.00	0.00±0.00	5.19±0.11*	20.71±0.13[#]	37.11±0.29[§]	50.60±0.49[&]
	pH 8.5	0.00±0.00	0.16±0.07	7.00±0.29*[ƙ]	23.75±0.25[#α]	50.31±0.42[§ʒ]	70.45±0.18[&ֆ]
	ASC, pH 7.4	0.00±0.00	0.00±0.00	0.00±0.00*	0.00±0.00[#ϙ]	3.19±0.31[§ɠ]	9.27±0.42[&ɘ]
	ASC, pH 8.5	0.00±0.00	0.11±0.09	0.99±0.15[ƙ]	5.21±0.12[αʂ]	8.83±0.11[ʒɲ]	12.62±0.11[ֆʋ]
RIF/ PENs	pH 7.4	0.00±0.00	0.00±0.00	0.00±0.00	4.12±0.24[#ϙʁ]	4.19±1.28[§ɥ]	3.77±0.37[&ɘɠ]
	pH 8.5	0.00±0.00	0.00±0.00	0.00±0.00	5.46±0.58[αʂɵ]	20.48±0.25[ʒɲ]	42.40±1.74[ֆʋφ]
	ASC, pH 7.4	0.00±0.00	0.00±0.00	0.00±0.00	12.50±1.18[#ϙʁ]	24.17±2.75[§ɠɥ]	44.63±4.68 [ɘɠ]
	ASC, pH 8.5	0.00±0.00	0.00±0.00	0.00±0.00	1.09±0.08[αʂɵ]	3.22±0.10[ʒɲ]	21.69±3.07[ֆʋφ]

n = 3, Mean ± Standard Deviation, Asterisks describe p value<0.05

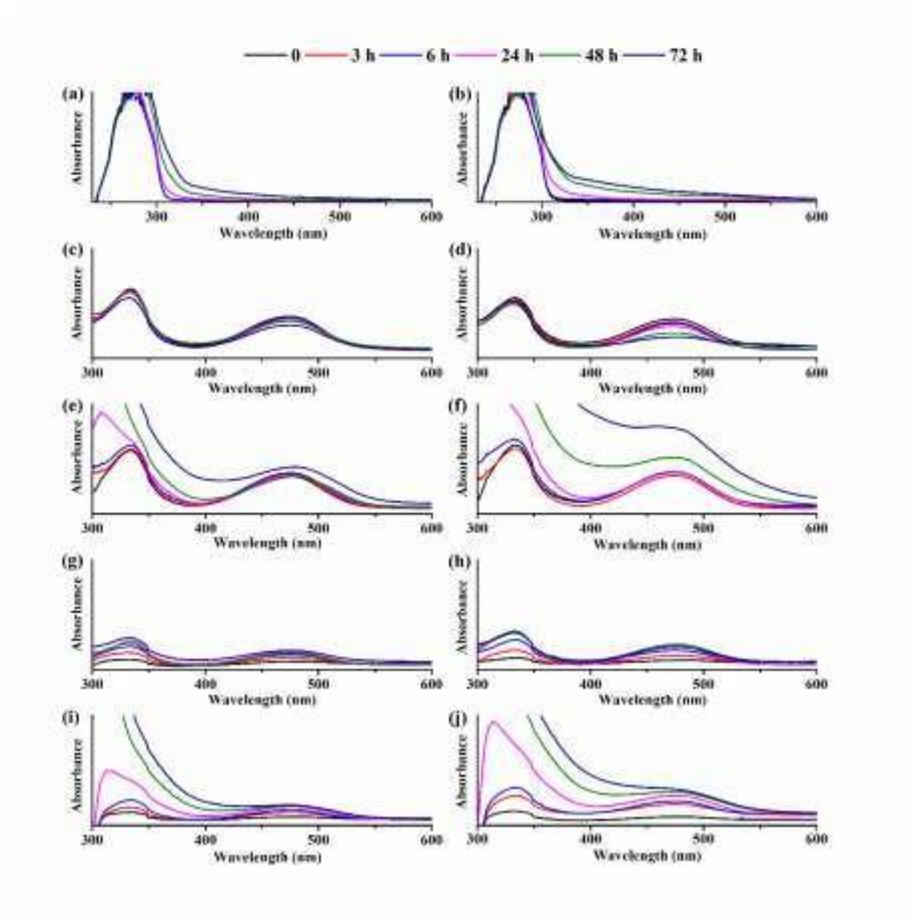

Figure S1. UV/Vis spectra of ASC/pH 7.4 (a), ASC/pH 8.5 (b), RIF/pH 7.4 (c), RIF/pH 8.5 (d), RIF/ASC/pH 7.4 (e), RIF/ASC/pH 8.5 (f), RIF/APENs/pH 7.4 (g), RIF/APENs/pH 8.5 (h), RIF/APENs/ASC/pH7.4 (i) and RIF/APENs/ASC/pH 8.5 (j).
